# Surgical Removal of a Calcified Right Atrial Mass Related to Venous Access port: A Case Report

**DOI:** 10.1016/j.ijscr.2020.03.043

**Published:** 2020-03-31

**Authors:** Joseph G. Brungardt, Anup K. Kasi, Jeffrey B. Kramer

**Affiliations:** a The University of Kansas, Department of Surgery, 4000 Cambridge, M/S 2005, Kansas City, KS 66160, United States; b The University of Kansas, Department of Oncology, 4000 Cambridge, Kansas City, KS 66160, United States; c The University of Kansas, Department of Cardiothoracic Surgery, 4000 Cambridge, Kansas City, KS 66160, United States

**Keywords:** Atrial mass, Port-a-cath, Cardiotomy, Thrombus, Case report

## Abstract

**Introduction:**

Intracardiac masses pose a difficult diagnostic and therapeutic dilemma. Indwelling catheters can lead to thrombus calcification causing untoward sequelae.

**Case presentation:**

We report on a patient who presented after computed tomography identified a large calcified right atrial mass. Her history included treatment for rectal cancer and breast cancer, thus we feared the mass could represent metastasis. The intracardiac mass was successfully resected via a right atriotomy. Her postoperative course was uneventful and the histopathology revealed a calcified thrombus.

**Discussion:**

In this report we discuss our findings and pre- and intraoperative considerations, as well as suggestions for management of implantable venous catheters. This is a rare complication of an indwelling catheter.

**Conclusion:**

Operative management of intracardiac lesions is the standard of care. When related to implantable catheters, the best patient care would be prevention of such lesions. This would include routine flushing of the indwelling catheters and prompt removal once not in use.

## Highlights

• Thrombus associated with catheters can cause untoward outcomes.
• Indwelling catheters must be cared for and undergo timely removal.
• Open heart surgery may be required to remove a mass inside the heart caused by these catheters.

## 1 Introduction

Intracardiac masses prompt expeditious and thorough workup and treatment by physicians. In some cases, extirpation of the mass utilizing cardiotomy will become necessary. Given the highly invasive nature of cardiac surgery, avoidance of an open-heart operation in an otherwise healthy patient would be ideal. Occasionally, intracardiac thrombi associated with indwelling vascular devices can be the cause of a mass, and proper management of these catheters could potentially obviate the need for an operation. It is difficult to estimate the number of patients discharged from a hospital with a central venous catheter inserted peripherally or with totally implantable ports. The risk of thrombosis of these lines is not insignificant and could result in serious, potentially fatal embolization into the pulmonary circuit or hinder blood flow across the right-sided tricuspid valve. Therefore, prevention of these scenarios may entail removal of these indwelling devices before large thrombi can develop associated with them. This work was reported in line with the SCARE criteria [1].

## 2 Presentation of case

A 50-year-old woman was referred to our academic tertiary care center for evaluation of a right atrial cardiac mass incidentally identified on positron emission tomography during second opinion consultation for her oncologic history. This patient’s history included breast cancer, treated 20 years prior with mastectomy and reconstruction, as well as rectal cancer, treated 10 years prior with neoadjuvant 5-fluorouracil followed by resection and adjuvant chemotherapy with 5-fluorouracial and oxaliplatin. The patient’s port was never removed. She received her oncologic care at an outside facility. She denied symptoms such as chest pain, syncope, shortness of breath, or palpitations. With such a history, we were concerned her cardiac mass could represent metastatic neoplasm. Were this to be a neoplasm, a more extensive operation would be necessary for resection and reconstruction. The patient was placed on an anticoagulant after our team’s discovery of this mass.

Prior surgical history included the above oncologic operations and an abdominal wall hernia treated with a mesh repair. She had never used tobacco and had no cardiovascular risk factors. She denied any personal or family history of venous thromboembolism or hypercoagulable disorders. Physical examination was only remarkable for prior surgical scars and trace lower extremity edema. Vital signs were normal. Her cardiopulmonary exam was normal. At baseline, labs were normal and electrocardiogram showed normal sinus rhythm.

**Fig. 1:**
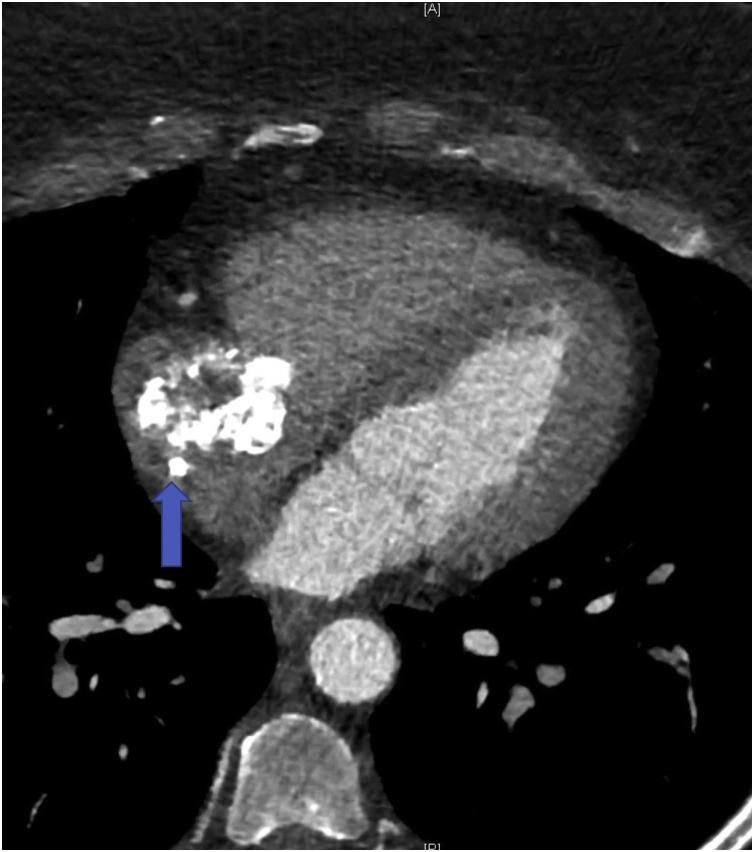
CTA coronary revealing the calcified right atrial mass and tip of the associated catheter (arrow).

The mass identified on recent imaging correlated, in retrospect, with a mass present on imaging 5 years prior, though it had grown during that interval from 3 cm in diameter to 4 cm. Further oncologic workup was negative for recurrence of her prior neoplasms. A CT angiogram of the chest revealed no evidence of atherosclerotic cardiovascular disease, but again demonstrated the mass (Fig. 1). Transesophageal echocardiography showed normal function and no clear attachment of the mass to the tricuspid valve, however it did appear adherent to a port-a-cath placed 6 years prior to this presentation. The limitations of imaging included exact extent and depth of atrial wall involvement. We also could not definitively determine the extent of catheter involvement.

Our differential of the patient’s mass included calcified thrombus and metastatic neoplasm. We felt a primary cardiac tumor was unlikely considering her history of prior neoplasms and long-term indwelling catheter. Given the proximity to the catheter and appearance on imaging, it seemed most likely to be calcified thrombus.

The patient had been referred to the cardiothoracic surgery service by her oncologist after discovery of this mass. Initially, percutaneous, transcatheter removal of this mass was contemplated however it was felt that this approach carried significant risk of partial or total embolization of a mass of that size. Thus, she was recommended to undergo surgical removal, to which she agreed.

**Fig. 2:**
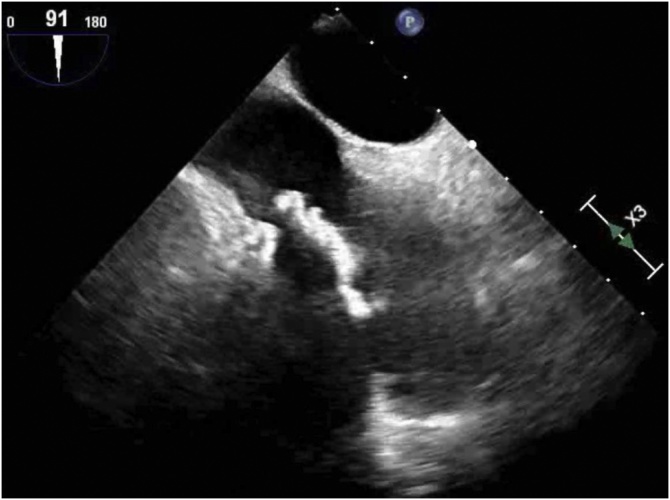
The mass in the patient’s right atrium intraoperatively by echocardiography.

Intraoperative transesophageal echocardiography confirmed the previously known details of the mass which appeared to be adherent to the catheter (Fig. 2). A median sternotomy was performed with cannulation of the ascending aorta. Venous cannulation included direct superior vena caval cannulation and an inferior cannula placed just below the diaphragm via the right femoral vein. The mass was in the proximity of the right atrial-inferior vena cava junction, precluding direct intrathoracic inferior vena cava cannulation.

**Fig. 3:**
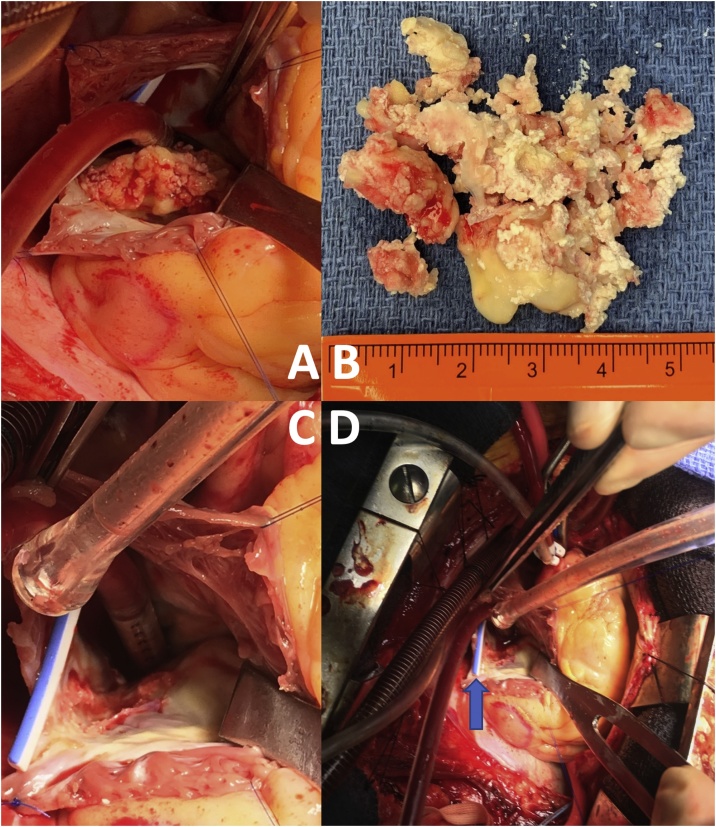
The mass during and after resection with a ruler for size. A. The calcified mass after right atriotomy. B. The mass after resection. C. Detail of right atrial wall after resection. The blue portion of the catheter is the patient’s right side. D. Resected right atrial mass with superior vena cava cannula, snare around inferior vena cava, cardiotomy suction, and white and blue port catheter exposed (arrow).

Upon entering the right atrium, it was apparent that the mass was densely adherent to the lateral wall of the right atrium but free of the catheter. The mass was found not to be attached to the tricuspid valve (Fig. 3). Considering her oncologic history, we considered performing a resection of the atrial wall with the mass, which would have required a patch reconstruction. A frozen section biopsy of the mass however revealed no malignancy, so instead the mass was shaved off of the atrial attachment (Fig. 3), and the atriotomy was closed. Although there remained a chance that malignant cells could in fact be present, we felt the likelihood of this was low, given the appearance of the mass, the ease that it separated from the right atrial wall, and the benign frozen section diagnosis. After separating from cardiopulmonary bypass, the tricuspid valve appeared to have mild to moderate regurgitation, consistent with her preoperative echocardiogram. The chest was closed in standard fashion and the port was removed.

The final pathologic review of the mass showed thrombus, a fibrin rim, and heavy calcification. The patient had an uneventful postoperative course and was discharged home on the fourth postoperative day. She was doing well on her 1-month postoperative visit with a healing incision and a return to her baseline cardiopulmonary examination.

## 3 Discussion

A variety of intra-cardiac tumors are well-described [2,3]. Primary tumors are somewhat rare, metastatic disease somewhat more common. In this instance, the tumor represented a benign, heavily organized intracardiac thrombus. Thrombi are typically treated with systemic anticoagulation, although on occasion either lytic therapy or catheter-based retrieval can be necessary, especially if there is presumed to be high potential for embolization, mainly due to increased size or configuration. Occasionally, “minimally invasive” techniques are inadequate, as was the case here, requiring direct excision of the mass in question. When the thrombus organizes there is a propensity to calcify, although few cases of calcified intracardiac thrombus have been reported [[4], [5], [6], [7], [8]]. These calcified thrombi were resected in an open approach and demonstrate involvement of a variety of cardiac chamber positions. Causative factors contributing to this include long term indwelling catheters and a hypercoagulable state, often related to a neoplastic processes. The common element though is the presence of a prothrombotic state, either from artificial material within the bloodstream or an existing prothrombotic disorder. Although they are often asymptomatic, intracardiac thrombi can obstruct the normal egress of blood from the heart or embolize, with resultant downstream problems. The evaluation of this consists of computed tomography, echocardiography, and, occasionally, magnetic resonance imaging. One management option could be watchful waiting for select intracardiac masses, especially those which are asymptomatic and chronic, as in this case, but many patients and surgeons prefer resection to prevent complications.

Totally implantable venous access devices (ports or port-a-caths) are placed for a variety of indications: durable long-term intravenous access for frequent hospital admissions, central delivery of chemotherapy, total parenteral nutrition, among others. Maintenance for these implanted ports typically consists of heparinized saline, a general consensus, though saline and thrombolytics have also been investigated as alternatives. Our institution’s maintenance, after completion of chemotherapy, includes flushing with heparinized saline every month and removal after 12 months following completion of chemotherapy administered for curative intent. General indications for removal included infection, bacteremia, and catheter related thrombosis. The overall incidence of all complication range from 3% to 66% for percutaneous central venous catheters but seem to be closer to 5–18% for implanted catheters [[9], [10], [11], [12]]. Elective port removal rates vary widely by institution and provider. Some physicians advocate routine removal, and some do not, with the thought that future chemotherapeutic interventions could be necessary at some point.

## 4 Conclusion

Although this report is anecdotal, the outcome in this case argues that strong consideration should be given to timely, elective removal of implantable catheters once intravenous chemotherapeutic treatment has been completed, particularly if patients have a reasonable life expectancy. If further treatment is required in the future, another device can be inserted at that time. Though these devices are relatively safe for long-term use, on occasion they can pose potential dangers to the patient, as well as lead to otherwise avoidable procedures and unnecessary angst.

## 5 Patient perspective

“I am very thankful for the medical teams that have been involved in my care. If it hadn't been for the 2nd opinion, I never would've even known about this mass. The surgeon made me feel at ease even though we had a rough start. I was a bit frustrated about all of this and how long it had been there. But again, very thankful that I was able to have a full recovery. I hope this will help other folks.”

## Declaration of Competing Interest

None.

## Sources of funding

None.

## Ethical approval

This is non-human subjects research and exempt from approval out our institution.

## Consent

The patient gave written consent for publication.

## Author contribution

All authors contributed equally to this manuscript. Dr. Brungardt drafted the paper. Drs. Kasi and Kramer edited and contributed significantly to the intellectual content. Dr. Kasi edited photography. Dr. Brungardt submitted the paper and compiled the supporting paperwork. The patient wrote the patient perspective.

## Registration of research studies

1. Name of the registry: n/a.
2. Unique identifying number or registration ID: n/a.
3. Hyperlink to your specific registration (must be publicly accessible and will be checked): n/a.

## Guarantor

Joseph G. Brungardt, Anup K. Kasi, Jeffrey B. Kramer.

## Provenance and peer review

Not commissioned, externally peer-reviewed.
